# Clinicopathological characteristics and survival of malignant fibrous histiocytoma of the bone: A population-based study using the SEER database

**DOI:** 10.1371/journal.pone.0232466

**Published:** 2020-06-03

**Authors:** Bo Liu, Hong Wei, Yan-Jun Ren, Debo Zou, Kaining Zhang, Qingwei Ma, Xing Xiao

**Affiliations:** 1 Department of Orthopedics, Linyi Central Hospital, Yishui, Shandong, People Republic of China; 2 Department of Orthopedics, The First Affiliated Hospital of Shandong First Medical University, Jinan, Shandong, People Republic of China; Universite de Nantes, FRANCE

## Abstract

Malignant fibrous histiocytoma of the bone (MFH-B) is an extremely rare and aggressive malignancy. The clinicopathological characteristics and prognosis of patients with MFH-B have not been defined. We conducted a retrospective study using the data of all MFH-B patients from the Surveillance, Epidemiology and End Results (SEER) database between 1975 and 2016. Initially, the clinicopathological characteristics were described. The difference in prognosis between patients with MFH-B and those with osteosarcoma was compared using propensity score matching analysis. Then, the features affecting the prognosis of patients with MFH-B were further determined using Cox regression analysis. A total of 318 patients with MFH-B were identified. The median overall survival (mOS) of all 318 patients with MFH-B was 29.0 months. The 1-, 3-, 5-, and 10- year survival rates were 67.4%, 53.6%, 38.7%, and 28.7%, respectively. The multivariate Cox regression analysis showed that older age, distant metastases, and flat bone lesion were independent factors for worse prognosis, whereas surgery was an independent factor for favorable survival, and this intervention could decrease risk of death by 61% (HR = 0.39, 95% CI 0.28–0.54). Apart from this, the prognosis of patients with MFH-B was significantly worse than that of patients with osteosarcoma in both unmatched and matched cohorts. In conclusion, MFH-B is a rare malignant bone cancer, with relatively worse prognosis than osteosarcoma. Older age, distant metastases, flat bone lesion, and surgery were independently associated with prognosis. In order to understand this disease more thoroughly and accurately, more cases with adequate information are required in the future.

## Introduction

Malignant fibrous histiocytoma (MFH), also known as undifferentiated pleomorphic sarcoma (UPS), is a pleomorphic tumor consisting of fibroblasts, myofibroblasts, and histiocytes, which is initially identified as a histologically distinctive type of sarcoma in 1964 [1, 2]. Since the characteristics of MFH were defined, this tumor has become the most common type of soft tissue tumor. The first case of MFH of the bone (MFH-B) was described in 1972, and MFH-B is an extremely rare and aggressive malignancy representing <1% of all primary malignant bone tumors [3]. MFH-B is a matrix-producing malignant tumor with a pleomorphic spindle-cell structure, which is devoid of any specific pattern of histologic differentiation [4]. MFH-B often occurs in the bone diaphysis or metaphysis that results in invasive bone damage and soft tissue mass.

MFH-B often exhibits an aggressive behavior associated with a high metastatic potential and high frequency of local recurrence. The principles of management of MFH-B are somewhat similar to those of osteosarcoma. A combination approach involving surgical resection and neoadjuvant chemotherapy is considered the mainstay of treatment. Patients with favorable necrosis (>90% necrosis) has a longer survival than those with inferior necrosis (<90% necrosis) [5, 6]. Some studies reported that MFH-B has a lower chemosensitivity than osteosarcoma although they showed similar prognosis [6]. And previous case series or case reports showed that age, metastatic disease, response to chemotherapy, and surgical resectability were associated with the prognosis of MFH-B.

However, knowledge of MFH-B is currently limited to case reports and small case series. The clinicopathological characteristics and survival in this type of cancer have not been well defined. Therefore, we performed a retrospective analysis of patients with MFH-B registered in the Surveillance, Epidemiology and End Results (SEER) database to describe the clinical characteristics and prognosis of patients with MFH-B to delineate features influencing prognosis.

## Materials and methods

### Participants

The SEER Program is the United States comprehensive cancer registry, with coverage of 30% of the US population, which recorded information of affected patients on a per case level including demographic characteristics and survival data [7]. We accessed the SEER database with the dedicated software SEER*Stat (http://seer.cancer.gov/seerstat) and obtained all available cases of primary MFH-B identified with ICD-O-3 histology coding (ICD-O-3: 8830/3) between 1975 and 2016, irrespective of the tumor localization. In each case, age at diagnosis, sex, localization distribution, TNM, SEER historic stage, radiation, chemotherapy, and surgery were recorded. Prognosis information was also isolated from the SEER database. The present study did not require ethical consent, because the SEER data were analyzed anonymously and were publicly available. In addition, all authors have signed the research agreement form and received permission from SEER to access the database.

### Statistical analysis

The distribution of MFH-B characteristics was presented using counts and percentages, and differences were determined using chi-square test for categorical data or t-test for continuous data. To evaluate the difference in overall survival (OS) between MFH-B and osteosarcoma, a propensity score matching (PSM) analysis was performed with a 1:1 ratio based on age, race, SEER historic stage, T stage, N stage, M stage, and surgery, radiotherapy, and chemotherapy [8]. Survival analysis was assessed using the Kaplan-Meier method, and the results were compared with the log-rank test. Univariate and multivariate Cox regression models were utilized to assess the association of each variable with prognosis. All statistical analyses were performed with SPSS 23.0 (SPSS Inc., Chicago, IL, USA). A P-value <0.05 was regarded as statistically significant.

## Results

A total of 318 patients with MFH-B were identified from the SEER database between 1975 and 2016. The demographic feature and clinical characteristics are presented in Table 1. The lesions of most MFH-B patients were located in the long bone (206/318) and lower limb (184/318). The lesion distribution was almost evenly divided by sex and laterality. Of all 318 included patients, 100 had localized stage, 114 had regional stage, and 64 had distant stage. Three of 92 patients were diagnosed with lymph node metastases, and 15 of 97 patients had distant metastases. Regarding the treatment regimen, a majority of patients with MFH-B (229/318) underwent surgery intervention, and 145 and 97 patients were treated with chemotherapy and radiotherapy, respectively. As shown in Table 1, a considerable amount of information were lacking including pathological differentiation, TNM stage, the use of radiotherapy and chemotherapy.

**Table 1:** Characteristics of 318 patients with malignant fibrous histiocytoma of bone.

Characteristics	Number (%)
Number	318
Age (Year)	56.0±20.0
Gender	
Female	154 (48.4%)
Male	164 (51.6%)
Ethnicity	
White	273 (85.8%)
Black	26 (8.2%)
Other	19 (6.0%)
Pathological Differentiation	
Well	3 (1.7%)
Moderate	15 (8.5%)
Poor	53 (29.9%)
Undifferentiated	106 (59.9%)
Unknown	141
Summary Stage	
Distant	64 (23.0%)
Regional	114 (41.0%)
Localized	100 (36.0%)
Unstaged	40
Laterality	
Left	124 (51.7%)
Right	116 (48.3%)
Unknown	78
Primary Site	
Long Bone	206 (66.6%)
Short Bone	4 (1.3%)
Flat Bone	58 (18.8%)
Irregular Bone	41 (13.3%)
Unknown	9
Primary Site	
Lower Limb	184 (57.9%)
Upper Limb	24 (7.5%)
Other	110 (34.6%)
Tumor stage	
T1	44 (57.1%)
T2	27 (35.1%)
T3	6 (7.8%)
T4	0
Unknown	241
Lymph Node Metastases	
N0	89 (96.7%)
N1	3 (3.3%)
Unknown	226
Distant Metastases	
Yes	15 (15.5%)
No	82 (84.5%)
Unknown	221
TNM stage	
I	4 (6.0%)
II	43 (64.2%)
III	3 (4.5%)
IV	17 (25.3%)
Unknown	251
Surgery	
Yes	229 (73.2%)
No	84 (26.8%)
Unknown	5
Radiation	
Yes	97 (30.5%)
No/Unknown	213 (69.5%)
Chemotherapy	
Yes	145 (45.6%)
No/Unknown	173 (54.4%)

**Fig 1:**
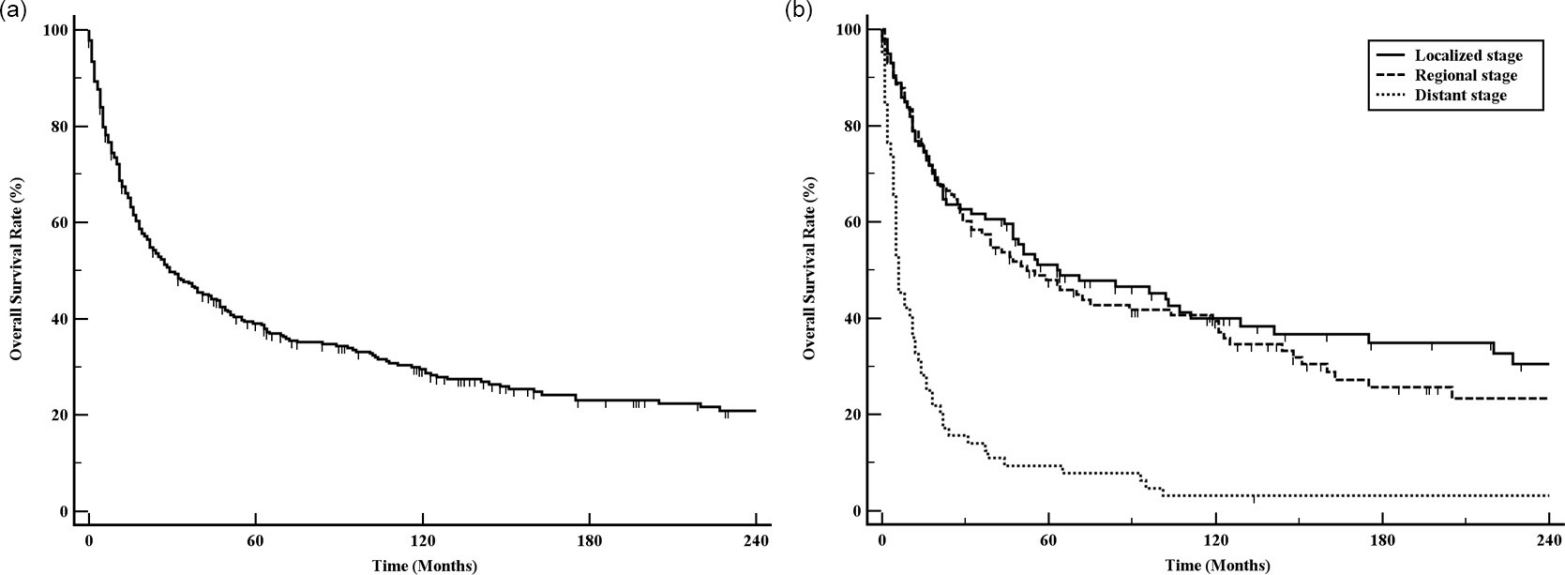
Overall survival for patients with MFH-B patients. Overall survival for 318 patients with MFH-B patients (a); overall survival for MFH-B patients with different SEER historic stage (b).

The median overall survival (mOS) of all 318 patients with MFH-B was 29.0 months (Fig 1A). The 1-, 3-, 5-, 10-, and 20-year survival rates were 67.4%, 53.6%, 38.7%, 28.7%, and 20.1%. Kaplan-Meier curves for survival stratified by SEER historic stage A classification are presented in Fig 1B. Patients with distant stages had significantly poorer prognosis than those with localized or regional stage (P*<*0.01 for both), only with a 7.8% of 5-year OS and 6 months of mOS (95% CI, 5.0–11.0) (Fig 1B). However, patients with MFH-B with regional stage had similar trend in OS with those with localized stage (P>0.05).

**Fig 2:**
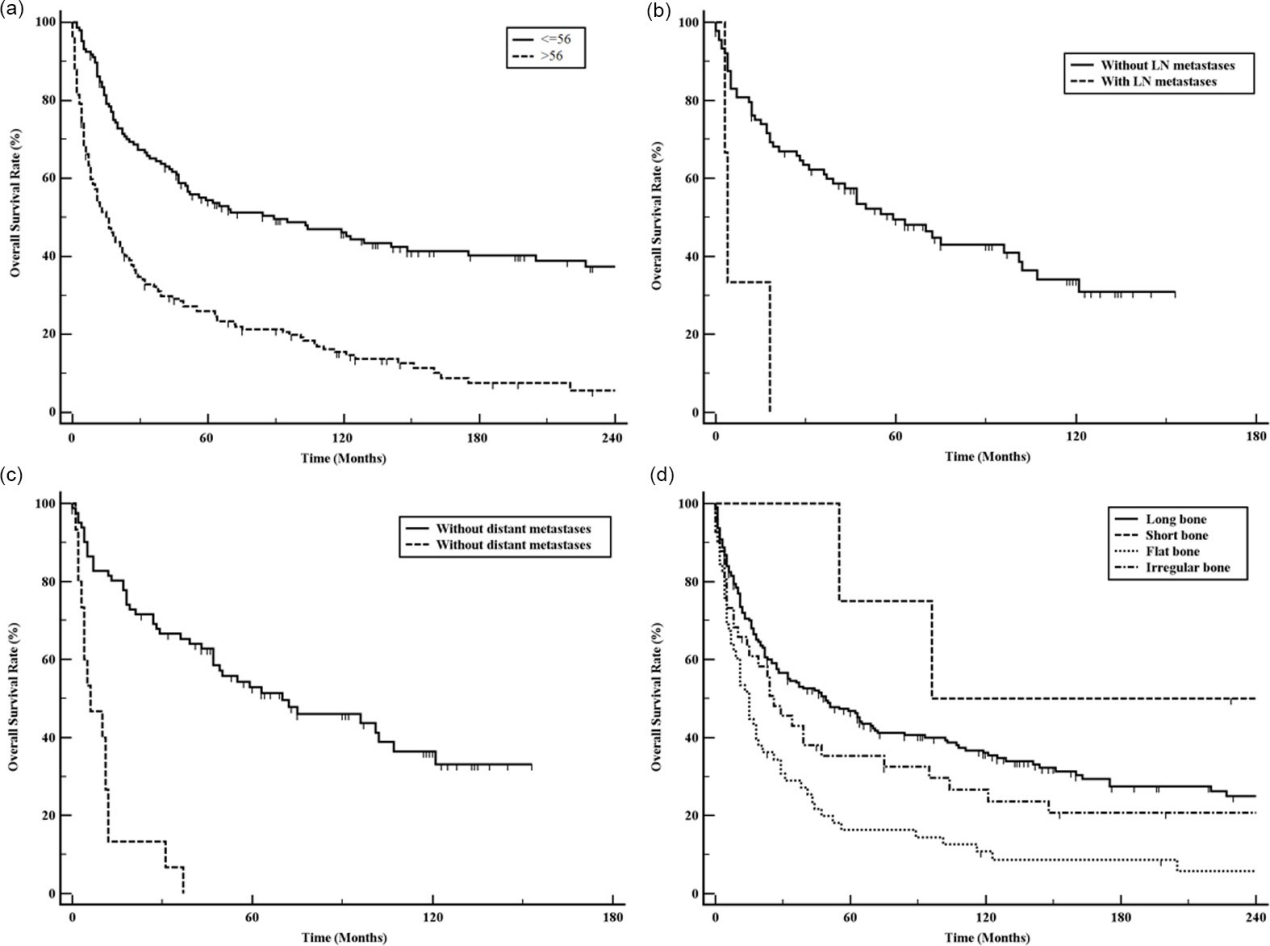
Overall survival for patients with MFH-B patients stratified by features. Age (a); lymph node metastases (b); distant metastases (c); lesion location (d).

The prognosis of patients with MFH-B worsened with increasing age (P<0.01) (Fig 2A). Younger patients (≤56 years) have significantly better prognosis than older patients (>56 years). The prognosis of these patients also worsened with invasion of tumor stage (T stage), but no significant difference could be observed among these patients (P = 0.09). The prognosis of patients with MFH-B with metastases was worse than those without metastases in both lymph node and distant metastases (P<0.01 for both; Fig 2B and 2C). Patients with lymph node metastases only had an mOS of 6.0 months (4.0–18.0 months), while the mOS of patients with distant metastases was only 4.0 months (3.0–11.0 months). The lesion location could also affect the prognosis of patients with MFH-B (Fig 2D; P<0.01). The survival analysis showed that patients with flat bone lesion had the worst prognosis. Apart from this, no significant association of other variables and survival could be observed: sex (P = 0.31), laterality (P = 0.09), race (P = 0.48), and pathological grade (P = 0.31).

**Fig 3:**
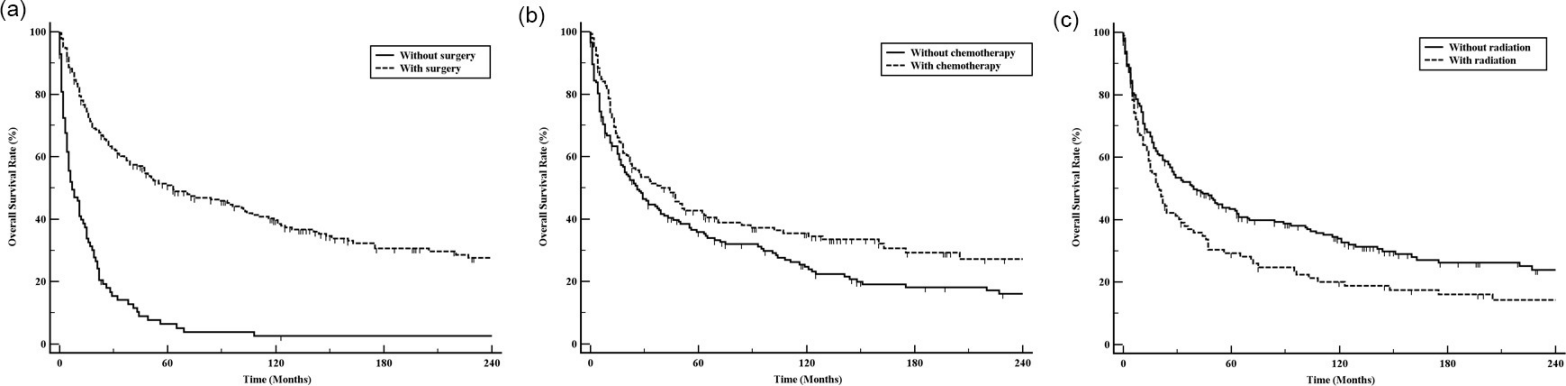
Influence of treatment regimen on overall survival for MFH-B patients. Surgery (a); chemotherapy (b); radiotherapy (c).

There is an immeasurably large difference in survival between patients who underwent and those who did not undergo surgical treatment (P<0.01; Fig 3A). The mOS of patients with MFH-B undergoing surgery was 63 months (95% CI, 47.0–102.0), while patients who did not undergo surgery only had an OS of 7 months (95% CI, 5.0–13.0). Chemotherapy could significantly prolong the mOS of patients with MFH-B by almost 13.0 months (39.0 months vs. 26.0 months; P = 0.02; Fig 3B). Conversely, patients who received radiotherapy had inferior outcome than individuals who did not receive radiotherapy (P<0.01; Fig 3C). Patients who received radiotherapy were more likely to have advanced stage (regional and distant 65% vs 52%) and less likely to undergo surgery (37.1% vs 21.7%) and chemotherapy (63.9% vs 50.2%).

Further, we performed univariate and multivariate Cox survival analyses to identify those variables that could potentially influence prognosis of patients with MFH-B based on the data in the present cohort of MFH-B patients. As shown in Table 2, older age, distant stage, metastases, flat bone lesion, and radiotherapy were significantly associated with poor prognosis, while surgery and chemotherapy contributed to good outcome (P<0.05 for all, Table 2). Multivariate Cox regression analysis showed that older age, distant metastases, and flat bone lesion were independent factors for worse prognosis in patients with MFH-B, whereas surgery was an independent factor for favorable survival, and this intervention could decrease risk of death by 61% (HR = 0.39; 95% CI, 0.28–0.54).

**Table 2:** Univariate and multivariate Cox proportional hazard analyses of clinical characteristics for overall survival rates in patients with malignant fibrous histiocytoma of bone.

Factor	Category	N	Univariate		Multivariate	
			HR (95% CI)	p-value	HR (95% CI)	p-value
Age	< = 56	151	Reference		Reference	
	>56	167	2.67 (2.03–3.50)	<0.01	2.50 (1.86–3.37)	0.06
Gender	Female	154	—	—	—	—
	Male	164	1.14 (0.88–1.47)	0.32	—	—
Race	White	273	Reference		—	—
	Black	26	0.92 (0.57–1.46)	0.72		
	Other	19	1.35 (0.80–2.28)	0.26	—	—
Pathological Differentiation	Well/Moderate	18	Reference			
	Poor /Undifferentiated	159	1.77 (0.95–3.29)	0.07	—	—
Summary Stage	Localized	100	Reference			
	Regional	114	1.15 (1.83–1.59)	0.40		
	Distant	64	4.01 (2.80–5.73)	<0.01		
Laterality	Left	124	Reference			
	Right	116	0.77 (0.57–1.04)	0.07		
Primary Site	Long Bone	206	Reference			
	Short Bone	4	0.46 (0.11–1.84)	0.27	0.69 (0.17–2.82)	0.61
	Flat Bone	58	2.08 (1.52–2.85)	<0.01	1.59 (1.13–2.25)	0.01
	Irregular Bone	41	1.39 (0.95–2.01)	0.08	1.61 (1.07–2.43)	0.02
Tumor Stage	T1	44	Reference		Reference	
	T2	27	1.37 (0.73–2.58)	0.33	1.69 (0.88–3.23)	0.11
	T3	6	2.76 (1.05–7.28)	0.04	1.62 (0.58–4.55)	0.36
Lymph Node Metastases	No	89	Reference		Reference	
	Yes	3	4.71 (1.71–15.1)	0.01	2.70 (0.76–9.56)	0.12
Distant metastases	No	82	Reference		Reference	
	Yes	15	4.91 (2.71–8.88)	<0.01	3.69 (1.89–7.31)	0.01
Surgery	No	84	Reference		Reference	
	Yes	229	0.27 (0.20–0.36)	0.04	0.39 (0.28–0.54)	0.01
Radiation	No/unknown	213	Reference		Reference	
	Yes	97	1.45 (1.11–1.89)	<0.01	0.90 (0.70–1.30)	0.79
Chemotherapy	No/unknown	173	Reference		Reference	
	Yes	145	0.74 (0.57–0.96)	0.02	1.08 (0.81–1.44)	0.59

**Fig 4:**
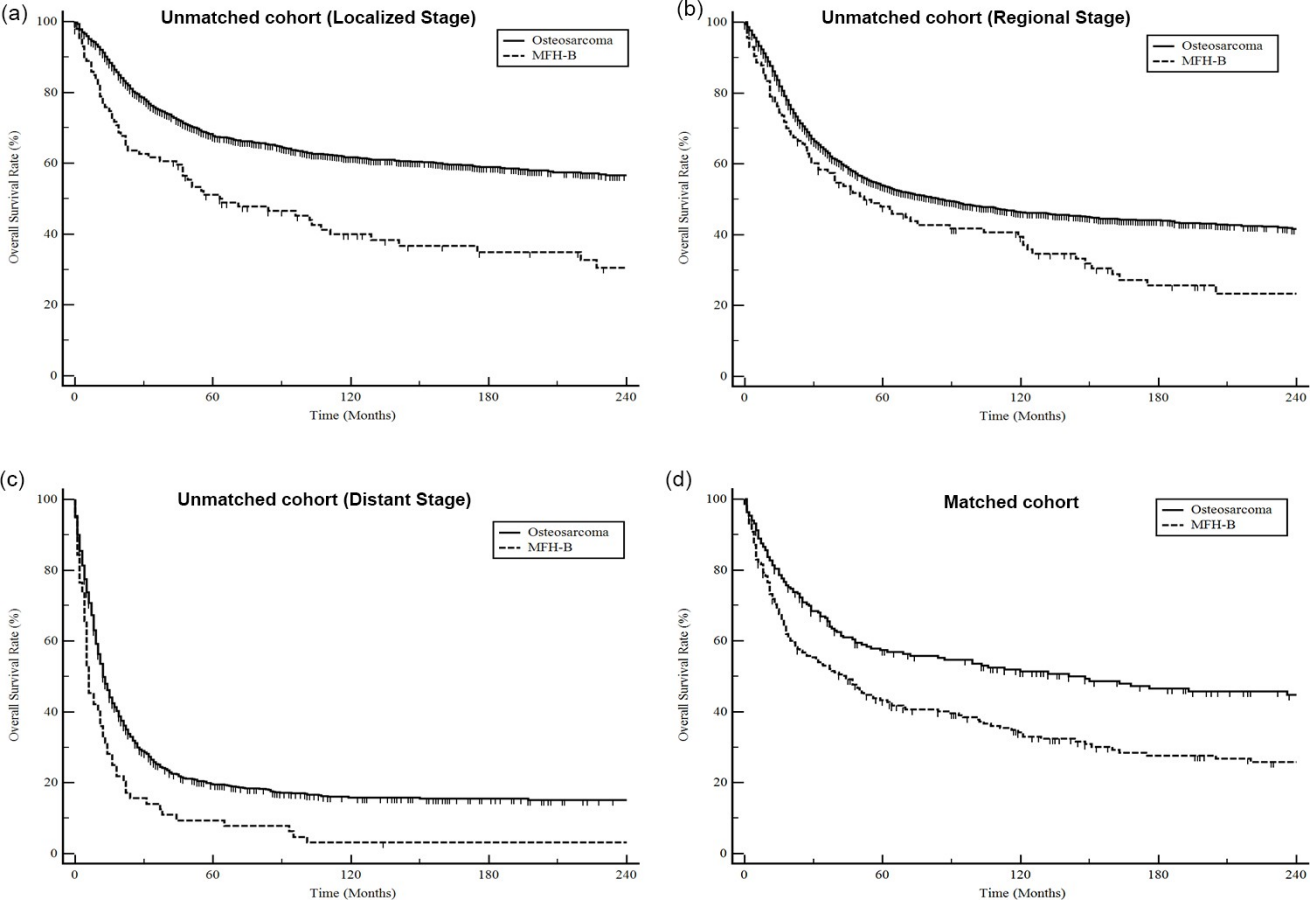
Comparative analysis of OS for MFH-B patients and osteosarcoma patients. Patients with localized stage (a); regional stage (b); distant stage (c) in unmatched cohort; in matched cohort after PSM analysis (d).

Further, we compared the difference in survival between patients with MFH-B and those with osteosarcoma. A total of 4840 patients diagnosed with osteosarcoma were identified from the SEER database between 1975 and 2016. In the unmatched population, patients with MFH-B had significantly poorer survival than patients with osteosarcoma, regardless of tumor stage (P<0.05 for all; Fig 4A–4C). To balance the characteristics and treatment regimens between patients with osteosarcoma and those with MFH-B, we performed a PSM analysis with 1:1 ratio based on age, sex, ethnicity, pathological differentiation, SEER stage, primary site, surgery, radiotherapy, and chemotherapy. A total of 217 patients with MFH-B were matched with 217 patients with osteosarcoma. No significant difference in clinical characteristics could be observed (S1 Table). As shown in Fig 4D, the survival analysis demonstrated that the prognosis of MFH-B patients was significantly worse than osteosarcoma patients in the matched cohort (mOS, 148 months vs 44 months; HR = 1.67; 95% CI, 1.31–2.12; P<0.01).

## Discussion

MFH-B is a relatively rare primary malignant neoplasm developing in the bone. The clinicopathological characteristics and prognosis for this disease are not fully defined. The present study aimed to analyze clinicopathological characteristics and prognosis of patients with MFH-B. We extracted the data of 318 patients with MFH-B from the SEER database to describe the clinicopathological characteristics and treatment regimen in the real-world study and identify the factors that could affect survival among this cohort.

Papagelopoulos et al. reported that the peak incidence of MFH-B was in the fifth to seventh decades of life, with a predominance in men of approximately 3:2 [9]. Another study reported that the average age at diagnosis varies from 34 to 51.5 years [10]. In the present cohort of 318 MFH-B patients, the average age was 56.0 years with a male:female ratio of 1.06:1. These data showed that MFH-B was relatively more common in younger men. A previous study reported that up to 54% of MFH-B cases developed at the knee joint, and the skeletal distribution of MFH-B favored the ends of the long tubular bone, typically the femur and tibia [11]. Flat bones tend to be less commonly affected, with reported rates for the pelvis ranging from 10% to 12% [12, 13]. This skeletal distribution of MFH-B corresponds to the findings of other large series. Our series showed that a majority of lesions (64.8%) were located in the long bone, followed by flat and irregular bones. As one of the highly aggressive tumor, our data also showed that almost all patients (159/177) with MFH-B had poorly differentiation or undifferentiation pathological grade. This pathological data further support the high malignancy of this rare cancer. Accumulated evidences demonstrated that MFH-B often has an aggressive behavior associated with a high rate of local recurrence and metastatic potential. A previous study found that 46% of patients with MFH-B could develop distant metastases. Moreover, the most common distant metastasis site was the lung (>80%), followed by the bone and regional lymph node [4, 10, 14]. In the present study, 23.0% and 41.0% of patients were classified as having distant and regional stages, respectively, according to the SEER historic stage. From the available information, distant metastases were found in 15.4% (15/97) of these patients. The proportion of lymph node metastases seems to be lower than those in previous studies. These differences may be ascribed to inadequate information that 226 patients had no information on lymph node metastases. In the present study, most patients (273/318) had white race, which is consistent with the race distribution in the United States. Similar to the data from the US population, a previous study on 56 Chinese patients with MFH-B reported that the median age was 38 years and 52.3% of patients were male with 68.2% of lesions located in the long bone and metastatic rate of 27.5% [15]. All these data suggest that patients with MFH-B may share similar clinical features among different ethnicities.

The median survival in MFH-B is 29 months, and 28.7% of patients could have survival of > 10 years. The 1- and 5- year survival rates are 67.4% and 38.7%, which are relatively lower than the 1- and 5-year survival rates of 89.0% and 53.0%, respectively, reported by Koplas [11]. Similarly, Bielack et al. performed a retrospective EMSOS study on 125 patients with MFH-B and obtained a 5-year disease-free survival of 59% [10]. Surgery and chemotherapy are considered the mainstay of treatment. In the present study, we observed that cancer-directed surgery significantly improved mOS in patients with MFH-B by almost 56.0 months. The multivariate Cox regression analysis showed that only surgery is an independent prognostic factor for good prognosis. Similarly, Natarajan demonstrated that the Kaplan-Meier 5-year survival rates of patients with MFH-B treated without chemotherapy and with chemotherapy after limb salvages surgery were 50% and 76%, respectively [3]. Consistent with these data, the present study also showed that chemotherapy could significantly prolong OS in patients with MFH-B. However, we could not evaluate the effect of neoadjuvant or adjuvant chemotherapy on OS due to inadequate corresponding data in the SEER database. Apart from this, the univariate analysis, but not the multivariate analysis, showed that patients receiving radiotherapy had lower survival than those not receiving radiotherapy. The main reason for these results is that patients receiving radiotherapy are more likely to have advanced stage and less likely to undergo surgery and chemotherapy, which could result in a poor prognosis. Additionally, in the present study, the results showed that the prognosis of patients with MFH-B were significantly lower than that of patients with osteosarcoma in both unmatched and matched cohorts. One explanation for this is the difference in the response to neoadjuvant chemotherapy. Picci et al. evaluated the analogies and differences in neoadjuvant chemotherapy between MFH-B and osteosarcoma and observed that MFH-B had a statistically significantly lower rate of good histologic response (27% vs 67%) [5]. With respect to histologic response to chemotherapy, MFH-B has a lower chemosensitivity than osteosarcoma, but a previous study showed that patients with MFH-B and those with osteosarcoma have similar prognosis when treated with chemotherapy based on MTX, CDP, ADM and IFO [5]. Second, MFH-B is more common in older patients while osteosarcoma is more common in younger patients even though the both may occur in either age group. For example, 17 of 318 MHF-B patients and 1970 of 4840 osteosarcoma patients were younger than 18 years in the present study. So we used the man age (56 year old) as the age cutoff, not the defined age (18 year old). In matched cohort, there is no significant difference between MHF-B patients and osteosarcoma patients that the age of MFH-B patients was 49.6±19.2 year and the age of osteosarcoma patients was 48.6±22.3 year. We also performed the comparative analysis of OS in unmatched cohort based on age distribution. In unmatched cohort, no significant difference in overall survival could be observed among patients younger than 18 years, but osteosarcoma had significantly better survival than MFH-B among patients older than 18 years (Data not shown). Of course, due to smaller sample size and the retrospective nature of this study, this difference should be validated in the future.

Except for tumor stage, older age and lesion location were also significantly associated with prognosis. Similar to other types of cancer, younger patients had much better prognosis than older patients [16]. Among patients with MFH-B, 47.8% of those aged < 56 years could survive for > 10 years, which is significantly better than those in older patients. Although flat bones tend to be less commonly affected, the present study showed that these patients had worst prognosis. Adjusting for other confounding factors, the multivariate analysis also showed that flat bone lesion is an independent prognostic biomarker for poor prognosis. Although the exact mechanism is unknown, we speculated that surgical treatment efficacy in this entity may be different from other patients. Of course, this needs to be validated in a further study.

There are several limitations that required clarification for accurate interpretation of the results. First, a considerable amount of information were lacking, such as lymph node metastases, distant metastases, and TNM stage. For example, there were 241 patients with unknown tumor stage, 226 patients had no recorded lymph node metastases, and 221 patients had no information on distant metastases. Meanwhile, 251 patients lacked TNM stage information. To our best knowledge, the AJCC method is more commonly used in clinical settings, but the SEER summary stage has standardized and simplified staging to ensure consistent definitions over time, which could be used as an alternative marker to measure disease progression in the SEER database, but there are still 40 patients without SEER summary stage. This point limited our ability to describe reliability and accuracy of prognostic analysis. Second, previous studies have reported that the majority of MFH arise de novo within the bone and approximately 22–28% of patients with MFH-B develop secondary to pre-existing bone lesions, including those in Paget disease, bone infarct, and prior bone irradiation [13, 17]. Secondary MFH-B has also been associated with worse prognosis than that of primary MFH-B [11, 12]. However, we could not evaluate the frequency of secondary MFH-B and difference in prognosis between patients with primary and secondary MFH-B due to inadequate data from the SEER database. Lastly, responses to treatment and recurrence rates could not be determined using these data from the SEER database. All these need to be further explored in the future.

In the present study, we described the clinicopathological characteristics and survival of patients with MFH-B using a population-based approach to provide crude stratification of prognosis based on commonly identified variables. Older age, distant metastases, flat bone lesion, and surgery were independently associated with prognosis. Patients with MFH-B had worse prognosis than those with osteosarcoma. This is the largest series to discuss clinical characteristics and outcome for patients with MFH-B, and these results are vital to disease management and future prospective studies for this rare cancer. Of course, in order to understand this disease more thoroughly and accurately, more cases with adequate information are required in the future.

## Supporting information

S1 Checklist (DOC)

S1 Table Characteristics of MFH-B patients and osteosarcoma patients in matched cohort after PSM analysis. (DOCX)

S1 File Raw-data about characteristics of all participants. (XLSX)
