# Supplementary material for: Clinicopathological characteristics and survival of malignant fibrous histiocytoma of the bone: A population-based study using the SEER database
Source: PLoS One. 2020 Jun 3;15(6):e0232466. doi: 10.1371/journal.pone.0232466 (PMC7269251; doi:10.1371/journal.pone.0232466)
Supplement: S1 Table — (DOCX) [file pone.0232466.s002.docx]

**S1 Table.** Characteristics of MFH-B patients and osteosarcoma patients in matched cohort after PSM analysis;

| **Characteristics** | **Osteosarcoma** | **MFH-B** | **P** |
| --- | --- | --- | --- |
| Number | 217 | 217 |  |
| Age (Year) | 48.6±22.3 | 49.6±19.2 |  |
| Gender |  |  |  |
| Female | 106 | 100 | 0.63 |
| Male | 111 | 117 |  |
| Ethnicity |  |  |  |
| White | 186 | 178 |  |
| Black | 15 | 23 |  |
| Unknown | 16 | 16 | 0.40 |
| Pathological Differentiation |  |  |  |
| Well | 3 | 2 | 0.28 |
| Moderate | 7 | 8 |  |
| Poor | 40 | 33 |  |
| Undifferentiated | 66 | 78 |  |
| Unknown | 101 | 96 |  |
| Summary Stage |  |  |  |
| Distant | 78 | 75 | 0.11 |
| Regional | 94 | 92 |  |
| Localized | 23 | 28 |  |
| Unstaged | 22 | 22 |  |
| Primary Site |  |  |  |
| Long Bone | 140 | 138 | 0.45 |
| Short Bone | 0 | 2 |  |
| Flat Bone | 35 | 78 |  |
| Irregular Bone | 35 | 63 |  |
| Unknown | 7 | 6 |  |
| Primary Site |  |  |  |
| Lower Limb | 137 | 134 | 0.78 |
| Upper Limb | 16 | 13 |  |
| Other | 64 | 70 |  |
| Laterality |  |  |  |
| Left | 121 | 105 | 0.35 |
| Right | 60 | 67 |  |
| Unknown | 36 | 45 |  |
| Surgery |  |  |  |
| Yes | 167 | 50 | 0.66 |
| No | 44 | 163 |  |
| Unknown | 6 | 4 |  |
| Radiation |  |  |  |
| Yes | 40 | 46 | 0.47 |
| No/Unknown | 177 | 171 |  |
| Chemotherapy |  |  |  |
| Yes | 116 | 129 | 0.21 |
| No/Unknown | 101 | 88 |  |
